# Constructing a cell microenvironment with biomaterial scaffolds for stem cell therapy

**DOI:** 10.1186/s13287-021-02650-w

**Published:** 2021-11-22

**Authors:** Xiaotong Zhao, Qiong Li, Zhikun Guo, Zongjin Li

**Affiliations:** 1 Henan Key Laboratory of Medical Tissue Regeneration, Xinxiang Medical University, 601 Jinsui Road, Xinxiang, 453003 Henan China; 2 Department of Cardiology, Zhengzhou Seventh People’s Hospital, Zhengzhou, China; 3 Nankai University School of Medicine, 94 Weijin Road, Tianjin, 300071 China

**Keywords:** Engineered microenvironment, Biomaterials, Scaffold, Stem cells, Cellular therapy, Tissue regeneration

## Abstract

Stem cell therapy is widely recognized as a promising strategy for exerting therapeutic effects after injury in degenerative diseases. However, limitations such as low cell retention and survival rates after transplantation exist in clinical applications. In recent years, emerging biomaterials that provide a supportable cellular microenvironment for transplanted cells have optimized the therapeutic efficacy of stem cells in injured tissues or organs. Advances in the engineered microenvironment are revolutionizing our understanding of stem cell-based therapies by co-transplanting with synthetic and tissue-derived biomaterials, which offer a scaffold for stem cells and propose an unprecedented opportunity to further employ significant influences in tissue repair and regeneration.

## Introduction

With the ability of differentiation and self-renewing, the critical role of stem cells in the formation of organisms and tissue repair and regeneration has been highlighted [1–3]. In detail, the undifferentiated state of stem cells in various tissues, if necessary, they can undergo cell division, and then differentiate into specialized cell types through their symmetric and asymmetric division patterns. Meanwhile, mechanisms controlling stem cell fate are considered fundamental factors for tissue and organ development and homeostasis, which is also important for better application of stem cell-based therapies in vivo [4–7]. Given that stem cells are widely reported to be capable of secreting bioactive factors, exerting immune modulation and angiogenesis, stem cell therapy has been utilized to regenerate injured tissues in in vitro applications [8, 9]. From numerous published studies, stem cell therapies are progressively recognized as a critical building block in tissue regeneration, offering cures for a wide variety of diseases, such as diabetes, myocardial infarction, and inflammatory diseases [5, 10, 11]. However, many challenges limit the successful use of stem cell translation in clinical practice, such as low cell retention and engraftment and poor long-term maintenance of stem cell function [12–14]. Therefore, a supportive microenvironment is needed to regulate stem cell function by activating or potentiating intrinsic host repairment after cell administration.

Engineered, fully defined materials are synthesized to overcome these bottlenecks regarding stem cell-based therapies. Biomaterials, conjugated growth factors [15] and tissue-derived extracellular matrix (ECM) [16], with tunable biophysical and biochemical properties to maintain and enhance stem cell function, are means of survival and differentiation of transplanted cells [2, 17]. Meanwhile, studies have shown that with heightened impacts of engraftment and differentiation, engineered materials co-transplanted with stem cells can facilitate functional recovery and structural integrity, such as angiogenesis and electromechanical improvement, providing a favorable niche for tissue regeneration [12, 18].

Driven by these emerging engineered platforms, alterations in microenvironment-mediated stem cell fate may ultimately satisfy the therapeutic application of stem cells in the clinic.

Therefore, in this review, the importance of engineered strategies in the regulation of stem cell homeostasis and fate in vitro, as well as mimicking the tissue microenvironment in vivo*,* is highlighted*.* At the same time, engineered platforms that control microenvironmental parameters and regulate cell–cell or cell-ECM interactions to direct stem cell growth and engraftment are discussed. Additionally, we summarize recent research on an artificial niche consisting of biomaterials, conjugated growth factors, or tissue-derived ECM, which can be utilized to provide a favorable microenvironment to enhance therapeutic effects in clinical translational applications (Table 1).

**Table 1:** Strategies for both synthetic and tissue-derived scaffolds to regulate cell microenvironment

Modified microenvironment	Manufacturing/processing	Engineering strategies	Reference
**Interaction with matrix proteins**	**Biomimetic scaffolds**	e g	
Stem cell enhancement and engineering	- Natural biomaterials	- Alginate hydrogel	[56]
		Chitosan (CS)	[61]
Tissue engineering and regeneration		Hyaluronans (HA)	[64]
	- Synthetic biomaterials	- Nanoparticle	[60]
		Biomatrix collagen	[63]
		PEG-HA-RGD-based hydrogel	[62]
		RGD-alginate hydrogel	[71]
**Immobilization of soluble factors**	**Conjugated growth factors materials**	e g	
Stem cell enhancement		- CS/PGE_2_	[79]
Tissue repair and regeneration		Dextrin-rhEGF	[99]
Immunomodulatory	**Controlled release materials**	- CS-IGF-1C	[104]
Proangiogenesis		CS-NO hydrogel	[106, 109]
**Interaction with ECM proteins**	**Natural ECM scaffolds**	e g	
Stem cell enhancement	- Tissue-derived ECM	- Cartilage matrix	[116]
Tissue engineering and regeneration		Matrigel	[120]
		Decellularized placenta matrix scaffoldDecellularized placenta matrix scaffold	[124]
	- Stem cell culture system by coating ECM scaffolds	- Hypertrophic (HY) ECM	[123]
		Endogenous ECM	[127]
**Signaling feedback from modified niche**	**EVs scaffolds**	e g	
		- RGD-biotin hydrogels	[69]
EVs engineering		CS-NO hydrogel	[121]
Tissue regeneration		Collagen matrix	[129]
Stem cell differentiation		Chitosan (CS)	[135]

## Limitation of stem cell therapy

With the ability to differentiate into an unlimited supply of tissue or organ-specific cells, stem cells can circumvent immunologic rejection after transplantation and promote the development of cell-based therapies in the treatment of a variety of debilitating disorders. Although cell therapy offers promise to restore function to benefit clinical practice, undesirable therapeutic outcomes challenge the translation process. The main hurdles that result in this phenomenon of stem cell-based therapy in clinical translational applications are as follows [19, 20]. When most cells leak out of the tissue or are mechanically washed out following blood flow, low retention rates of administered cells can be a critical limitation [21, 22]. Meanwhile, exogenous stem cells in the hostile, ischemic and inflammatory microenvironment are unfavorable to survival and proliferation, which even can lead massive cells to die in vivo [23]. Furthermore, modest improvement in stem cells cannot completely change the notable up-regulation of related oxidative stress and harmful cytokines, as well as anoikic at injured sites in the complex and dynamic pathological environment [20, 24, 25]. Another bottleneck is that the use of stem cells at the clinical level was not as effective as expected, various ex vivo processes must be performed to achieve the desired results [26]. In the hope of contributing to stem cell-based treatment options, enhanced strategies are summarized to address the low retention and survival of implanted cells. Interestingly, bioengineering approaches may provide solutions to overcome the current limitations in stem cell translational applications.

## Cell microenvironments

**Fig. 1:**
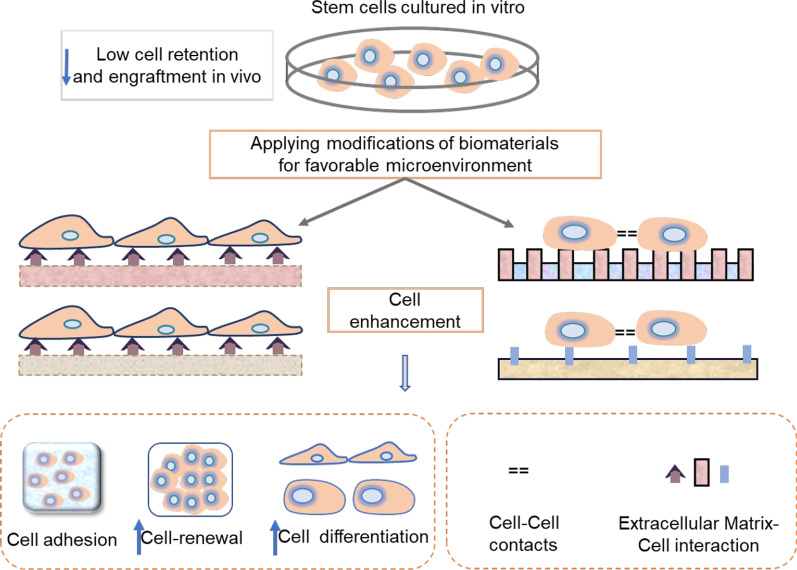
Engineered microenvironment for stem cell therapy. Low cell retention and engraftment exist in vivo transplantation of stem cells. To address these challenges, biochemical or biophysical modifications of biomaterials are urgently needed to establish a favorable microenvironment for stem cell therapy. By means of engineered bioactive scaffolds, a suitable niche was designed to trigger specification events of stem cell fate specification events, enhancing cell differentiation, retention, engraftment, and self-renewal in vivo via cell–matrix or cell–cell interaction, and thus promoting tissue repair and regeneration

Stem cells residing in specific anatomic locations in the body are also termed three-dimensional (3D) microenvironment or niches that include surrounding cells, ECM, secreted or bound biomolecules, and cytokines that are critical for their functional enhancement after transplantation [27]. The microenvironment as a dynamic system can trigger stem cell fate specification events such as quiescence, self-renewal, and differentiation, contributing to a wide variety of physiological stages of development, homeostasis, and responses to injury/stress [28–30]. However, a poor environment on the one hand can be the main obstacle that leads transplanted cells to exhibit poor cellular retention and engraftment in vivo. On the other hand, the delivery of stem cells into an ischemic and hypoxic environment generally leads to cellular apoptosis and further poor cell viability and engraftment that affected successful translational applications [31, 32]. Furthermore, damaged tissues often lose deeper layers, indicating that the use of engineered scaffolds is important in establishing functionalized artificial niches for stem cell-based therapy [33]. To address these challenges, advances in modulating stem cell behavior, viability, and retention are urgently needed [34]. As it can modulate and recapitulate these complex cellular architectures and properties, as well as biochemical and biophysical signals, mimicking the native microenvironment is essential to guide stem cell fate decisions and control cell behavior [35, 36]. Consequently, engineered bioactive scaffolds that mimic the niche-like 3D microenvironment are hopeful strategies by regulating stem cell fate and cell–cell or cell–matrix interactions and allowing to be degraded and remodeled, which will eventually enhance therapeutic efficacy [37] (Fig. 1).

## Strategies for cell enhancement

**Fig. 2:**
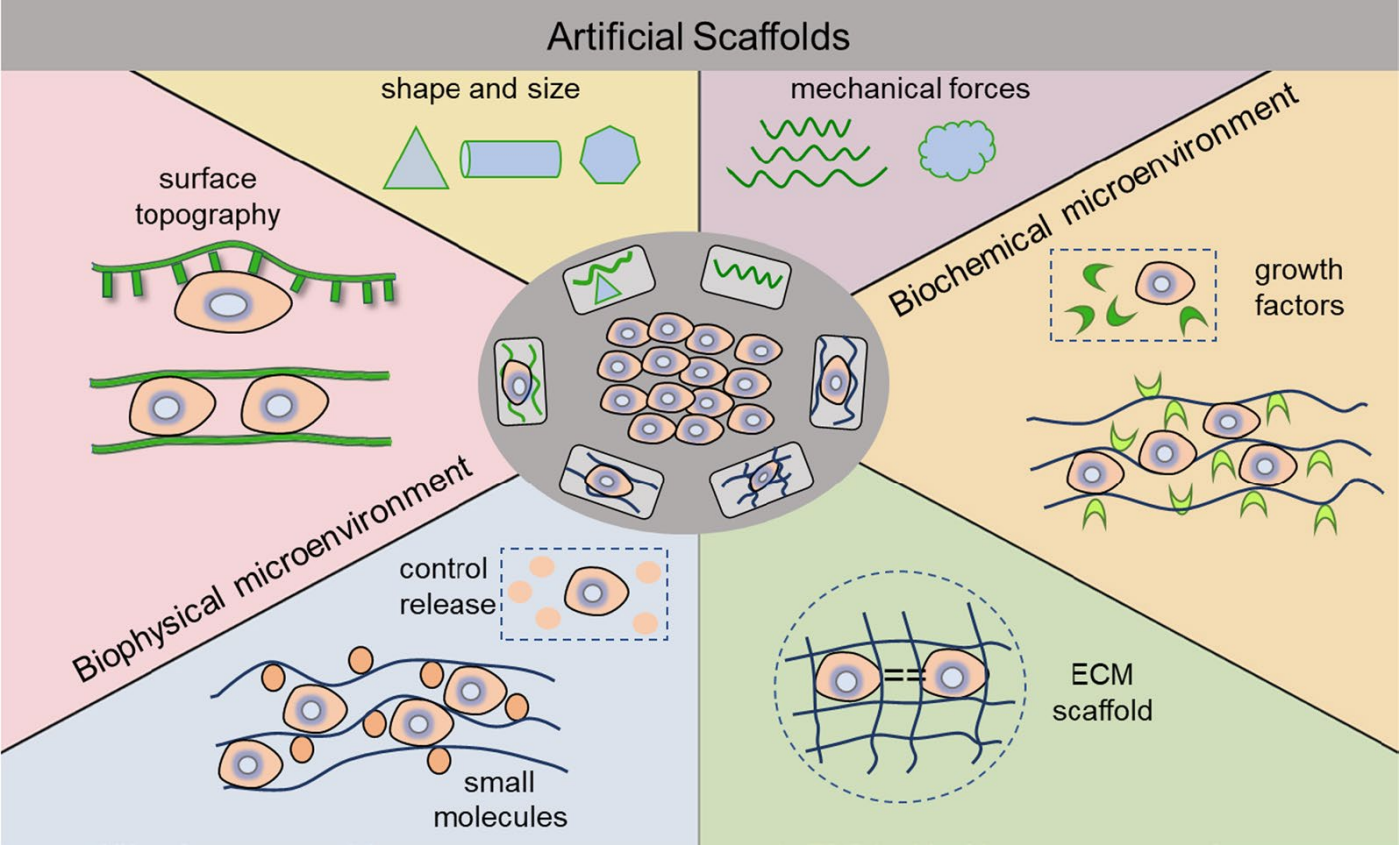
Strategies for artificial scaffolds for cell enhancement. Engineered biomaterials based on their biochemical and biophysical microenvironment, for example, the characteristics of biophysical cues of surface topography, material shape and size, and mechanical forces, as well as biochemical cues to conjugate growth factors, controlling to release specific small molecules and tissue-derived ECM scaffold, were extensively applied in stem cell-based therapy to improve cell maintenance in vitro and in vivo. Strategies designed to regulate cell behavior play an important role in enhancing therapeutic effects after transplantation in vivo

Recent advances in the field of scaffold-based developmental tissue engineering are expected to revolutionize stem cell-based therapy via enhancing cell function in vivo after transplantation. The concept of developmental engineering strategy was first proposed by Lenas et al. by using biomaterial scaffolds as cell support [38]. Developmental engineering that includes several characteristics such as robustness for process stability and product reproducibility to resist to varieties of external adverse factors, signaling pathway dependence for in vitro developmental processes emphasizing the subsequential developmental stage, semi-autonomous meaning for self-established conditions and progresses naturally according to its needed processes, can facilitate tissue growth and cell differentiation, and the formation of intermediate tissue with modular behavior [38]. In other words, the strategy represents “in vitro biomimetics in vivo tissue development”, emphasizing the process of in vitro tissue engineering. Meanwhile, the rules of biomimetic process design have been developed comprehensively to enable tissue engineering to become a technology-based discipline, which promoted the improvement of cell-based therapy by constructing a cell microenvironment based on engineering scaffolds [39]. In the process of bone healing, the treatment of large bone defects is still a challenge for the remaining complications after bone grafting. The developmental engineering strategy to mimic the natural healing cascade or promote stem cell differentiation for rapid bone regeneration is considered a promising application in biomedicine [40, 41]. In the application of cell enhancement, artificial scaffold parameters such as appropriate soluble and surface-bound cytokines, cell–cell interactions, ECM, physicochemical cues, and mechanical forces are required to control and regulate stem cell behaviors through cell–cell or cell–matrix adhesion interactions [42, 43]. Strategies for stem cell enhancement and controlled release systems at a certain site of the body are necessary for tissue repair and regeneration. Therefore, stem cells incorporated with engineered biomaterials, growth factors, or small molecules are absolutely imperative to boost the retention and survival rate of stem cells and further facilitate tissue regeneration in vivo (Fig. 2).

### Biomimetic scaffolds for stem cell therapy

Currently, biomaterials have spurred substantial advances to improve therapeutics at the clinical level, and at the same time, provide a favorable platform for the establishment of artificial niches to control stem cell culture and differentiation [2, 44]. On the basis of their components and structures, biomaterials are capable of transmitting specific signals on biochemical levels that satisfy the role of cells in the tissue repair process. Stem cells can interpret biomaterial instructions through cell–matrix interactions and then modulate their fate determination. To direct cell fate, several crucial parameters need to be considered within biomaterials, such as topography, chemistry, and physical properties. Several studies have reported that surface topography and chemical composition can regulate cell adhesion, differentiation, migration, and proliferation [45, 46]. Gradually, scaffolds containing beneficial characteristics were introduced to satisfy engineering approaches, which could activate specific biological responses and not change bulk properties [47–49]. As the mechanical properties of the substrate can control stem cell fates, bio-nanocomposites represent a fundamental role in the improved mechanical and functional properties of synthetic polymers [50, 51].

Moreover, some of the bio-nanocomposites could be modulated by introducing conductive nanostructures to exert electrical properties, such as carbon nanostructures (e.g., nanotubes, graphene, and nanofibers) and metal nanostructures (e.g., gold, silver). Results have suggested that the integration of two different nanostructures facilitates the development of bio-functional porous scaffolds that range from specific bioactivity, structural and mechanical integrity, to electrical conductivity. For example, Fortunati and Misra et al. developed a ternary nanocomposite scaffold, a novel biodegradable/bioactive composite material containing three different materials by incorporating multiwalled carbon nanotubes (MWCNT) [52, 53]. Furthermore, with respect to the properties of interconnectivity, pore size, and shape, scaffold morphology is another critical factor for stem cell-biomaterial interaction. The appropriate morphology of biomaterial scaffolds, especially their microstructures, is important for cell differentiation and tissue response. In essence, smart polymeric nano-systems with an appropriate composition can be defined as artificial scaffolds to mimic the morphologic structure and function of the surrounding tissue. At the same time, scaffolds should also be capable of enhancing cell functions, such as cell attachment, differentiation, maintenance, and migration, as well as autocrine production of growth factors, immunomodulators, and other bioactive factors [46, 54, 55].

Inspired by the better biophysical and biochemical properties of functional materials, integrative stem cells and engineering approaches are increasingly being utilized to address challenges in regenerative and translational medicine [56]. Compared to traditional two-dimensional (2D) culture, three-dimensional (3D) mesenchymal stem cell (MSC) culture exerts better multi-differentiation potential and can be considered an excellent culture system [57, 58]. In previous studies, thermosensitive hydrogels based on chitosan (CS), with their change from liquid to gelation at room temperature and excellent biocompatibility, are widely used as an ideal 3D injectable scaffold to deliver stem cells in the tissue repair and regeneration process [59, 60]. For example, the combination of chitosan-coated conduit and neurosphere cells with human adipose-derived stem cells (ADSCs) exhibited functional recovery in the sciatic nerve [61]. Similarly, the enhanced approach of the hyaluronic acid hydrogel scaffold to deliver ADSCs has shown a notable improvement in the treatment of burn wounds, which is due to its properties of improving neovascularization and wound closure, and reducing scar formation [62]. Abundant evidence has explained that biomaterial scaffolds can alleviate noxious insults and create nurturing and protective environments that augment the therapeutic efficacy of implanted cells [63, 64].

Biomimetic materials have been reported to serve as fully defined scaffolds or carriers to successfully deliver bioactive agents and therapeutic molecules for application in tissue engineering [65]. As stem cells require cell adhesion through cell–cell or cell-ECM interaction through integrin involvement to prevent anoikis, it is especially necessary to investigate integrin activation and cell adhesion in engineered biomaterials [66]. Studies have determined that the non-fouling native alginate has the characteristics of biocompatibility, high water content, tailor ability, and low cost, as well as easily modified to supply specific binding sites for cell adhesion. The hydrogel, an ideal 3D scaffold, contains specific cues for stem cell differentiation, reducing the inflammatory reaction after co-transplantation [67, 68]. In addition, a biofunctionalized scaffold of arginine-glycine-aspartic acid (RGD), a self-assembled peptide with a specific cell recognition motif, can bind strongly to the integrin of stem cells, triggering integrin-stimulated cell adhesion [69]. Tripeptide nanoparticles, as a widely used peptide sequence, could mimic the native niche by constructing a 3D structure environment [70]. Furthermore, a study on retinal pigmented epithelium (RPE) and neural retina generated from human embryonic stem cells/induced pluripotent stem cells (hESCs/hiPSCs) with RGD-alginate scaffolds also showed that it was useful to compensate for the current protocol and improved the formation of other pigmented, neural or epithelial tissue through the derivation, transportation and transplantation of neural retina and RPE [71]. Apparently, these findings initiate the survival and proliferative pathways of anchorage-dependent cells [72]. Engineered materials are ideal scaffolds for providing a biomimetic 3D system to support nano-biomaterial and stem cell interactions and to direct stem cell behavior [44, 73].

**Fig. 3:**
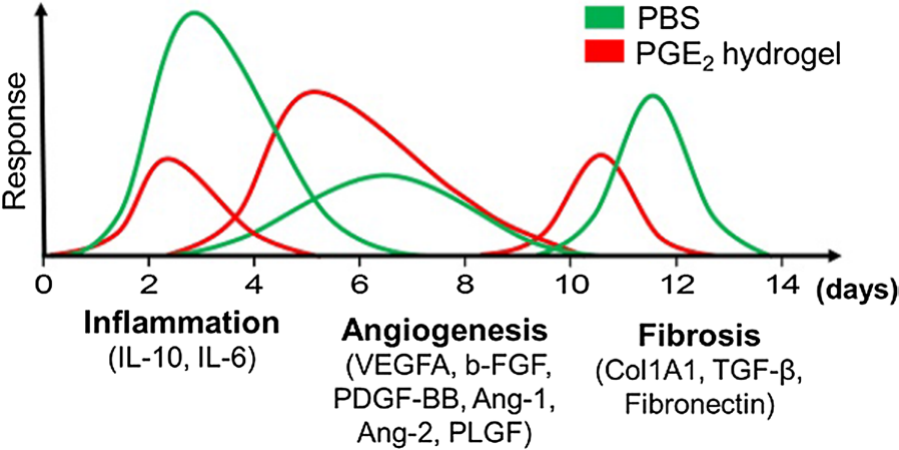
Incorporation of PGE_2_ into the CS hydrogel created a balanced microenvironment in vivo. Inflammation, tissue regeneration, and remodeling are three important phases in wound healing events at the injured site. In this study, the increased anti-inflammatory and pro-angiogenic activities of macrophages and the reduction of fibrosis were investigated, which demonstrated that the wound microenvironment was better improved by hydrogel in vivo, exhibiting a balanced niche of the overlapping inflammatory, regenerative (angiogenesis) and remodeling (fibrosis) phases of cutaneous wound healing. Reprinted with permission from [79]

In addition to the intrinsic regenerative, angiogenic, and tissue repair properties of stem cells, the immunomodulatory effect of innate and adaptive immune cells has been progressively investigated [74]. Influences of monocytes/macrophages on stem cell fate are considered important factors when stem cells are delivered to poor surroundings of diseased or injured tissues. Therefore, determining the immunophenotype of monocytes/macrophages among encapsulated stem cells is crucial for immune cells, which can be changed by absorbed proteins on the surface of the biomaterial by affecting their adhesion, apoptosis, pro- and anti-inflammatory cytokines, such as the secretions of ECM proteins and growth factors [75, 76]. Furthermore, a study has revealed that prostaglandin E_2_ (PGE_2_) incorporated into chitosan (CS) hydrogels can alleviate inflammation by promoting the transformation of M1 macrophages into the M2 phenotype, which can secrete high levels of interlukin-10 (IL-10) [77, 78]. Similarly, applying the incorporation of PGE_2_ and CS hydrogel to a murine cutaneous wound healing model exerted a better therapeutic effect, demonstrating the improvement of the niche at the injured site. Regulation of balanced cytokines and mediators in wound healing in vivo, especially the balance of three overlapping phases (Fig. 3) that include inflammation, regeneration (angiogenesis) and remolding (fibrosis), could guide tissue repair and regeneration [79]. Furthermore, a study of MSCs that entrap a specific macrophage immunophenotypes with the gelatin/polyethylene glycol-based matrix has indicated a considerable improvement in adipocyte differentiation and further promotes normal wound healing [80]. Advances in developmental engineering strategies with the capacity to construct a suitable microenvironment for stem cell-based therapy, protein replacements, or gene therapies may pave the way for some incurable diseases, such as rare genetic epidermolysis bullosa (EB) [81], and bone grafting [82].

The crucial role of immune cells involved in the tissue repair/regeneration processes after implantation or injury by secreting cytokines, promoting the degradation of ECM and clearing debris, can mediate the interaction between synthetic or tissue-derived biomaterials implantation and the cell microenvironment to orchestrate tissue regeneration. Therefore, the properties of biomaterial implants should be considered, such as appropriate physical, chemical, and biological signals [83, 84]. In detail, engineering biomaterials will recruit plasma proteins like fibronectin, vitronectin, albumin, and others on the surface after implantation, which are important factors to stimulate the adhesion and activity of immune cells [85]. Regarding the physical signals of implants, including shape, different surface properties, and substrate stiffness, increasing evidence demonstrated that the role of their immunomodulation properties mainly focused on macrophages by designing appropriate implants conditions to regulate the anti-inflammation response by the secretion of IL-4 and IL-10 (M2 subtype of macrophages) to meet the need for tissue restoration after injury [86, 87]. In terms of chemical signals, mounting studies have shown that the chemical composition of biomaterials, such as inorganic signals (metallic ions, ceramics), and functional groups, can be utilized and modified, and some of them can be released from biodegradable implants. These chemical signals can interact with immune cell adhesion and protein to control the production and release of cytokines in the process of immunomodulation in vivo [88, 89]. Biological factors such as genes, ECM, stem cells, and cytokines, as a straightforward way to influence immune responses, are regarded as an effective way for immunomodulation. The biomaterial network can be used as delivery system and support scaffold to regulate inflammation and tissue homeostasis [90, 91]. On the other hand, targeted immunomodulation strategies to mediate specific immune cells by providing a suitable microenvironment may drive the development of advanced tissue engineering and stem cell therapy.

### Biomaterials with growth factors for stem cell therapy

**Fig. 4:**
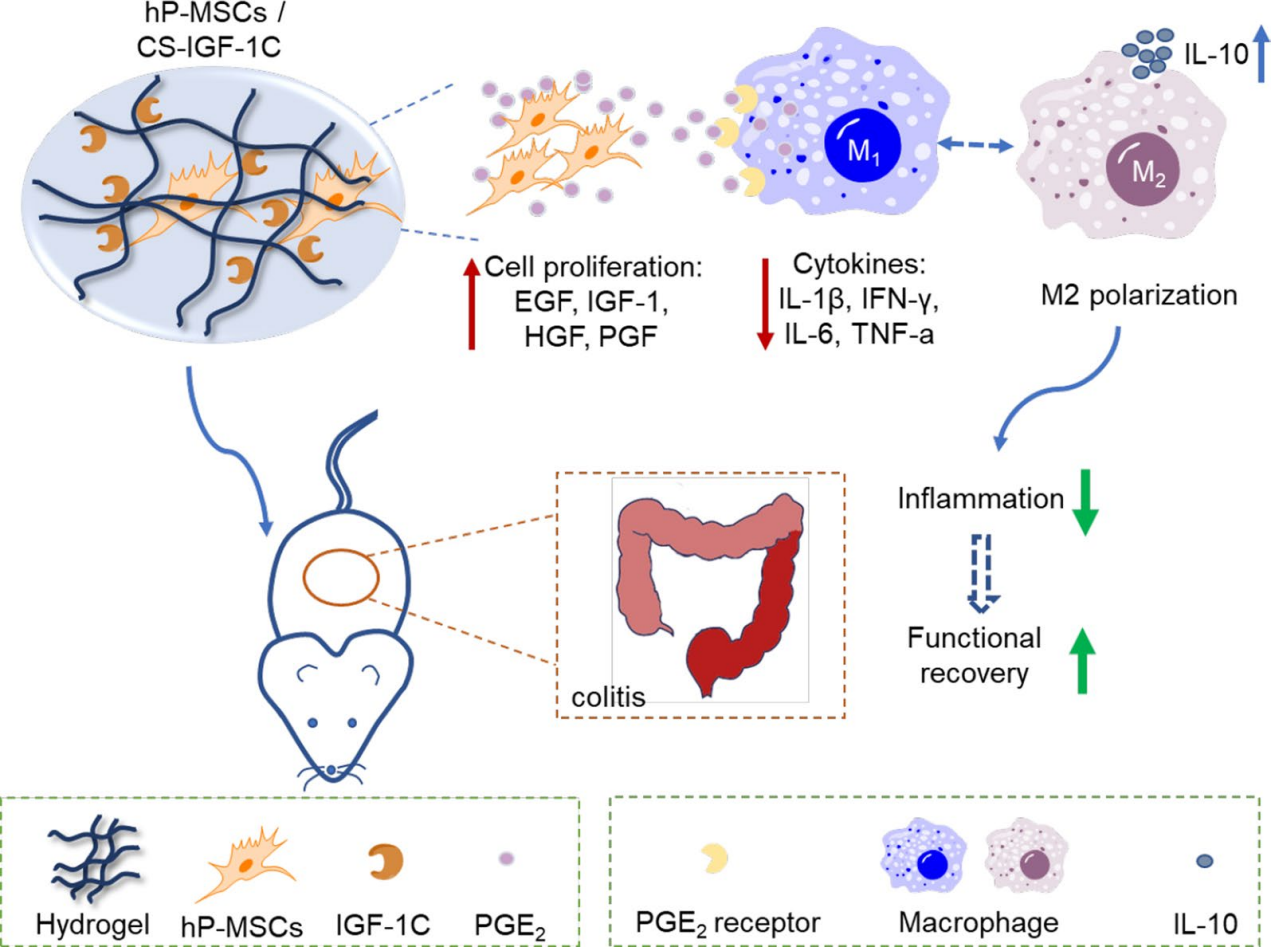
The engineering approach provides a niche for stem cell transplantation. A biomaterial designed with growth factors was applied for stem cell transplantation, for example, human placenta-derived MSCs (hP-MSCs) applied to treat trinitrobenzene sulfonic acid (TNBS)-induced colitis in mice [104]. First, stem cells were cultured in CS-IGF-1C hydrogels in vitro, stimulating the production of PGE_2_, and then upregulating the cell proliferation markers of EGF, IGF-1, and HGF. Second, stem cells were co-transplanted with the hydrogel in vivo and PGE_2_ released from stem cell uptake through binding to its receptor in macrophages. Down-regulated genes of IL-1β, IFN-γ, IL-6, and TNF-α were observed and the secretion of IL-10 by M2 macrophages, promoting the polarization of M2 macrophages in colitis of the mouse model. Finally, the results determined the functional recovery of colitis mice under the stem cell co-transplantation system of stem cells with conjugated growth factors

Growth factors can be used to support stem cell survival, proliferation, and differentiation by communicating with cells, which requires exogenous application in cell enhancement [92]. In the context of tissue repair and regeneration, the delivery of growth factors to adherent cells can stimulate and accelerate functional recovery of injured organs [93, 94]. Since exogenous growth factors are easily degraded in cultured medium, engineered biomaterials, such as hydrogels are suitable candidates for conjugation by means of proper chemistry, topography, and mechanics characteristics [95, 96]. In previous research, the conjugation of growth factors to bio-functional hydrogels that are capable of spontaneously eroding a physical microstructure on poly (vinyl alcohol) (PVA) was developed. Then, covalent immobilization of growth factors was employed to use as an efficacious means of communicating with adhering cells and thus improved cell proliferation [97]. To guide the neuronal differentiation of MSCs, a nerve growth factor conjugated to electrospun nanofibrous meshes with its topographic signals significantly promoted cell function after injury [98]. Recently, studies have determined that localized delivery of dextrin-conjugated growth factors for bioactive therapeutic agents can support stem cell expansion and differentiation as an adjunct to promote tissue repair [99, 100]. Meanwhile, the hybrid biomaterial scaffold with conjugated growth factors to deliver bone marrow-derived MSCs in vivo effectively promoted neovascularization and bone formation in organ repair [101]. Furthermore, considering the essential role of insulin-like growth factor-1 (IGF-1) in tissue regeneration, a short synthetic IGF-1C peptide was introduced and immobilized in chitosan hydrogel material (CS-IGF-1C) and then transplanted with ADSCs, indicating a significant improvement in functional recovery and structural integrity of injured organs [12, 102]. Similarly, the artificial microenvironment of the CS-IGF-1C scaffolds exerted a good influence on angiogenesis in mouse hindlimb ischemia [103]. Furthermore, better therapeutic effects of the CS-IGF-1C scaffold were observed in a mouse colitis model by activating MSC secretion of PGE_2_ [104], which can promote M2 macrophage polarization (Fig. 4). On the basis of the above, the development of innovative and effective biomaterial scaffolds with conjugated growth factors is expected to be an alternative strategy in promoting the development of regenerative medicine.

### Controlled release of small molecules for stem cell therapy

**Fig. 5:**
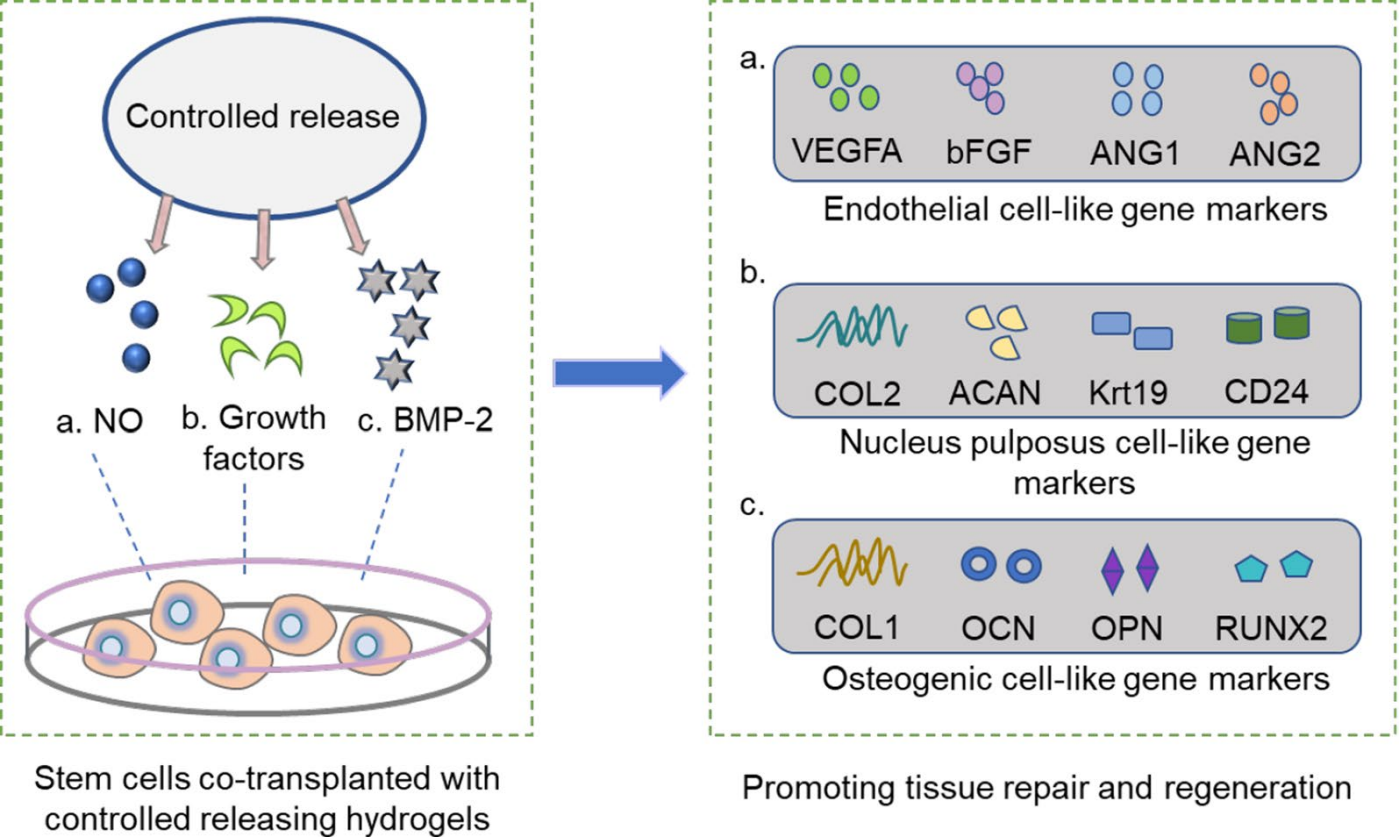
Controlled release of small molecules, growth factors, and proteins. Control release systems can be considered an effective way to modulate stem cell behavior. **a** The utilization of NO-releasing hydrogels to support stem cell delivery through the control of NO generation can upregulate the expression of endothelial cell-like phenotypes, such as VEGFA, bFGF, ANG1, ANG2, and then significantly facilitate neovascularization in mouse with ischemic hindlimb [109]; **b** Injectable GMs were employed to deliver growth factors and as vehicles of stem cells, which can promote cell differentiation into nucleus pulposus (NP)-like gene markers of COL2, ACAN, Krt19, CD24, determining a promising approach for the in vivo treatment of rat degenerative disc disease [111]; **c** The PLGA and SL deliver system were designed to control the release of BMP-2, and then applied the platform to build a suitable microenvironment for stem cell culture. In this culture system, stem cell matrix mineralization abilities were detected, and osteogenic cell-related gene expression of COL-1, OCN, OPN, and RUNX2 was demonstrated, indicating the potential of the engineered platform for bone tissue regeneration [110]

Currently, engineered substrates with controlled release of bioactive factors, proteins, or growth factors have been regarded as vehicles to offer a desirable niche for stem cell delivery, which can improve cell proliferation and engraftment and further improve the therapeutic efficacy of implanted cells [105, 106]. Nitric oxide (NO), a highly reactive radical, is an essential molecule to regulate cellular/molecular functions and physiological processes associated with tissue regeneration in mammals [107]. However, the short biological half-life of NO limited its application in stem cells [108]. In response to local NO release, a preliminary study of endothelial differentiation of mouse embryonic stem cells (ESCs) was observed when ESCs were incorporated in a CS-based hydrogel to release NO in a controllable manner. Clearly, NO release occurred only in the presence of β-galactosidase [21]. Similarly, stem cells with NO stimulation have also been shown to exert superior therapeutic effects by enhancing the proangiogenic potential in mouse hindlimb ischemia [109]. Meanwhile, to fully capture and control bone morphogenic protein-2 (BMP-2), a platform with nanoporous poly (lactide-coglycolide) (PLGA) microspheres mediated by soybean lecithin (SL) was developed to deliver stem cells and was ultimately determined to be significant for bone tissue regeneration [110]. Recently, a study showed that injectable gelatin methacryloyl (GelMA) microspheres (GMs) act as carriers to control growth factor release and as a delivery vehicle for transplanted stem cells to promote functional recovery of injured tissues in rat degenerative disc disease [111]. Collectively, stem cells are co-transplanted with bio-functional scaffolds that carry profitable bioactive factors, which can not only create optimized microenvironments to improve cell function, but also act as controllable release systems to compensate for the energy and nutrition deficit due to diseased conditions in the body and eventually facilitate tissue repair and regeneration (Fig. 5).

## Extracellular matrix derived from tissue for cell enhancement

The extracellular matrix (ECM), as one of the important microenvironment parameters of stem cells, is closely related to cell survival and fate. Mounting evidence has suggested that the ECM-based microenvironment is related to the biophysical properties and biochemical extracellular stimuli of stem cells and could determine the cell fate between self-renewal and differentiation [112]. Constitutively, it can be obvious that the limits of transplanted stem cells can be attributed to the loss of their excellent potential when taken outside of their niche, leaving them alone at injured sites where there is no functional vascular network to support cell survival [113, 114]. Advancing in the microenvironment based on engineered ECM has been gradually shown for stem cell therapy [115]. Additionally, the mechanical properties of biomaterial scaffolds, especially tissue-derived ECM is particularly crucial for a 3D cell culture system. With the characteristics of viscoelastic and stress relaxation, ECM has been widely applied in the field of tissue regeneration, such as cartilage tissue engineering, where the mechanical confinement of ECM regulates chondrocytes to form cartilage matrix to replace damaged cartilage. Simultaneously, the tunable mechanical properties of matrix scaffolds can enhance stem cell function, such as the capacity of activity and osteogenic differentiation [116–118]. Therefore, considering the significance of cell-ECM interactions in the engineering strategy for cell culture, the mechanical parameters of biomaterial scaffolds should be highlighted.

Matrigel, a reconstituted basement membrane, is considered a commonly used plate-coding matrix for controlling stem cell fate and a vehicle for cell administration in a variety of dimensions [119, 120]. Similarly, when human ESC-derived endothelial cells were suspended in Matrigel, the supplied substrate could considerably reverse the down-regulated expression of a series of ECM and adhesion molecule-specific genes [121]. The changes in these genes after cell detachment provided valuable information for the interaction between cell-ECM [122]. Furthermore, the strategy that uses the hypertrophic type of designed ECM scaffolds providing the necessary microenvironment of endochondral ossification by regulating stem cell functions is important for promoting osteogenic differentiation and repairing bone defect [123]. Recently, a study on decellularized extracellular matrix (dECM) isolated from perinatal tissues, especially placenta-derived ECM, has shown significant effects on anti-inflammatory and proangiogenic in skin wound healing. At the same time, this research identified the crucial role of glycosaminoglycans (GAG) in the treatment of wound injury [124], providing an encouraging substitute to employ the GAG-enriched placental dECM hydrogel for cell or drug delivery in the development of tissue regeneration.

Importantly, depending on the characteristics and properties of ECM scaffolds, it is of great importance to deliver meaningful soluble and immobilized factors in the determination of sophisticated extracellular signals such as self-renewal and lineage commitment [125, 126]. Preliminary research has shown that human amniotic fluid stem cells (hAFSCs) cultivated with endogenous ECM and adhesion molecules facilitate cell retention and are beneficial for cardiac function recovery when injected directly into the intramyocardial tract in a rat model [127]. Moreover, collagen, as an important component of ECM that relies on many biological activities and good mechanical properties, provides a favorable niche for stem cell growth. For example, a functional collagen matrix scaffold was used to improve stem cell function by promoting cell adhesion, proliferation, and migration for in situ tendon regeneration [128, 129]. Collectively, the benefits of an ECM-based niche that improves the survival and revascularization of administered stem cells are a promising strategy to be further developed.

## Engineering strategies for stem cell-derived extracellular vesicles

Extracellular vesicles (EVs) secreted from cells can carry vital information in the form of lipids, proteins, mRNAs, and small RNAs such as microRNAs (miRNAs) and can participate in intercellular communication [130, 131]. Recently, EVs as paracrine factors released from stem cells have been extensively utilized in varying animal disease models and are an alternative to stem cell-based therapy due to their therapeutic potential [132]. However, the retention and stability of EVs following time in vivo after transplantation can be a major obstacle in clinical reality [133]. Hopefully, after encapsulation and transplantation with engineering strategies, EVs can maintain their biological activity and achieve controlled release in vivo for a longer time [134]. When stem cell-derived EVs were incorporated with the CS hydrogel, the stability of miRNAs and proteins, as well as their retention, was significantly enhanced in vivo. Meanwhile, the abilities of pro-angiogenesis and endothelium-protective of engineered EVs were assessed in vitro, demonstrating better therapeutic effects for hindlimb ischemia [135]. Additionally, EVs released from nitric oxide-releasing polymer treated MSCs exhibited proangiogenic capacity both in vitro and in vivo by building a microenvironment for stem cell culture, which revealed superior tissue repair and functional recovery for ischemic disease [106]. In general, engineering strategies may facilitate the development of cell-free therapeutic applications, whether biomaterial scaffolds were incorporated with stem cell-derived EVs, or applied to cell culture systems.

## Translational application of biomaterials in stem cell therapy

Many effective strategies for stem cell therapy have been translated into clinical investigations as a result of considerable favorable supports in the regenerative capacity of stem cells [136]. Furthermore, increasing research has shown that stem cell transplantation has the potential to allow specific regeneration of injured or diseased tissue in patients [17]. Although with the transient presence of stem cells in a few days after transplantation, clinical investigations using stem cell products have exerted a significant tropic influence on immune and inflammatory responses after transplantation, especially the two successful applications of limbal stem cells for eye burns and MSCs for pediatric graft versus host disease [137]. Additionally, engineered platforms such as biomaterials, conjugated growth factors and small molecules, and tissue-derived ECM providing a favorable microenvironment for stem cell function are emerging as a means of approach for tissue regeneration in preclinical studies [129, 138]. Hence, driven by the increasing development of engineered platforms, the advancement of cell niches can elicit excellent properties of translational therapy, which is full of hope for improving disease prevention and treatment and eventually contributing benefits to patients.

## Conclusion

Regarding its significant properties on the paracrine secretion of bioactive factors, angiogenesis, and immunoregulation, stem cell-based therapy has potent therapeutic effects on diseases such as myocardial fraction, hindlimb ischemia, cutaneous wound, colitis, kidney, and spinal injury, as well as bone defect/graft and ocular burns, and thus can be considered a promising approach in regenerative medicine. Moreover, biomaterials and materials with conjugated growth factors and small molecules, as well as dECM matrix, have largely served as bioactive scaffolds to engineer a suitable niche to facilitate functional recovery of implanted cells and further enhance therapeutic efficacy in vivo. In conclusion, engineered platforms provide a favorable microenvironment to promote the maintenance and engraftment of exogenous cells. Meanwhile, stem cell therapy should attach more importance to the application of developmental engineering strategies, which provide excellent properties in improving the functional recovery and structural integrity of diseased organs.

## Data Availability

Not applicable.

## Abbreviations

ADSCs: Adipose-derived stem cells
BMP-2: Bone morphogenic protein-2
CS: Chitosan
CS-IGF-1C: Chitosan modified C domain of IGF-1
dECM: Decellularized extracellular matrix
EB: Epidermolysis bullosa
ECM: Tissue-derived extracellular matrix
ESCs: Embryonic stem cells
EVs: Extracellular vesicles
GelMA: Gelatin methacryloyl
GMs: GelMA microspheres
GAG: Glycosaminoglycans
hESCs/hiPSCs: Human embryonic stem cells/induced pluripotent stem cells
hAFSCs: Human amniotic fluid stem cells
IGF-1: Insulin-like growth factor-1
MSCs: Mesenchymal stem cells
NO: Nitric oxide
PGE_2_: Prostaglandin E_2_
PVA: Poly (vinyl alcohol)
PLGA: Poly (lactide-co-glycolide)
RGD: Arginine-glycine-aspartic acid
RPE: Retinal pigmented epithelium
SL: Soybean lecithin
